# TonEBP: A Key Transcription Factor in Microglia Following Intracerebral Hemorrhage Induced-Neuroinflammation

**DOI:** 10.3390/ijms25031438

**Published:** 2024-01-24

**Authors:** Ailiyaer Palahati, Yujia Luo, Le Qin, Yuhao Duan, Mi Zhang, Hui Gan, Xuan Zhai

**Affiliations:** 1 Department of Neurosurgery Children’s Hospital of Chongqing Medical University, National Clinical Research Center for Child Health and Disorders, Ministry of Education Key Laboratory of Child Development and Disorders, Chongqing Key Laboratory of Child Neurodevelopment and Cognitive Disorders, Chongqing 400010, China; 2021110408@stu.cqmu.edu.cn (A.P.);; 2 Center for Neuroscience Research, Chongqing Medical University, Chongqing 400016, China

**Keywords:** intracerebral hemorrhage, inflammation, TonEBP, NF-κB, PELI1

## Abstract

Transcription factors within microglia contribute to the inflammatory response following intracerebral hemorrhage (ICH). Therefore, we employed bioinformatics screening to identify the potential transcription factor tonicity-responsive enhancer-binding protein (TonEBP) within microglia. Inflammatory stimuli can provoke an elevated expression of TonEBP in microglia. Nevertheless, the expression and function of microglial TonEBP in ICH-induced neuroinflammation remain ambiguous. In our recent research, we discovered that ICH instigated an increased TonEBP in microglia in both human and mouse peri-hematoma brain tissues. Furthermore, our results indicated that TonEBP knockdown mitigates lipopolysaccharide (LPS)-induced inflammation and the activation of NF-κB signaling in microglia. In order to more deeply comprehend the underlying molecular mechanisms of how TonEBP modulates the inflammatory response, we sequenced the transcriptomes of TonEBP-deficient cells and sought potential downstream target genes of TonEBP, such as Pellino-1 (PELI1). PELI has been previously reported to mediate nuclear factor-κB (NF-κB) signaling. Through the utilization of CUT & RUN, a dual-luciferase reporter, and qPCR, we confirmed that TonEBP is the transcription factor of *Peli1*, binding to the *Peli1* promoter. In summary, TonEBP may enhance the LPS-induced inflammation and activation of NF-κB signaling via PELI1.

## 1. Introduction

Intracerebral hemorrhage (ICH), a severe form of stroke, causes roughly 2.8 million deaths annually across the world [1]. Survivors of ICH frequently experience impaired mobility and cognitive disorders due to the lack of specific treatments [2]. Primary brain injury, characterized by intracranial hypertension and neurological impairment, arises from extensive hematoma and mechanical compression [3]. In the hours following the ICH, secondary brain injury develops, causing tissue injury over the following days or weeks [4]. Multiple pathophysiological processes initiate and propagate secondary injury, such as the leakage of damage-associated molecular patterns (DAMPs), erythrolysis, microglial activation, and peripheral macrophage and neutrophil infiltration [5,6]. Activated microglia in the peri-hematoma region, which surrounds the hematoma in the brain in hemorrhagic stroke, secrete numerous inflammation-related molecules, including cytokines and chemokines, exacerbating edema and the infiltration of peripheral neutrophils and macrophages, thereby exacerbating neurological deficits [7,8]. There have been numerous studies demonstrating that transcription factors in microglia regulate the expression of pro-inflammatory cytokines [8]. To identify the key transcription factors in microglia that regulate inflammatory responses, we utilized bioinformatics to screen for tonicity-responsive enhancer-binding protein (TonEBP), a transcription factor associated with microglia-induced inflammation.

TonEBP was previously reported as an important hypertonic stress modulator in the renal medulla [9]. However, the critical role of TonEBP in many physiological or pathological processes, in an osmostress-independent manner, has been reported [10,11]. High-fat-diet-induced diabetes in mice results in increased TonEBP expression in the brain [12]. Furthermore, inflammatory triggers such as interferon-γ and interleukin-4 have been documented to augment TonEBP in primary microglia [13]. Within macrophages, TonEBP stimulates the induction of pro-inflammatory cytokines like interleukin (IL)-6 and IL-1β, polarizes M1 macrophages, and inhibits M2 alternation [14]. Additionally, the absence of myeloid TonEBP diminishes macrophage activation, enhances arginase-1 and IL-10 (M2 phonotype markers), and suppresses inflammation responses [10]. Consequently, TonEBP may play a role in neuroinflammation following ICH.

The nuclear factor-κB (NF-κB) complex is involved in a variety of cellular processes, including immunity, inflammation, tissue growth, survival, and apoptosis [15]. Under stimulation with damage-associated molecular patterns (DAMPs), the NF-κB signaling pathway is activated, subsequently activating pro-inflammatory microglia [16]. The NF-κB signaling boosts pro-inflammatory cytokines’ secretion in microglia, such as tumor necrosis factor (TNF)-α, IL-1β, and IL-6, thereby facilitating neuroinflammation [16,17]. In our current research, we discovered that TonEBP modulates lipopolysaccharide (LPS)-evoked inflammation and NF-κB signaling. However, the mechanisms through which TonEBP regulates inflammatory responses warrant further investigation. In this work, our team conducted RNA-seq to identify a novel downstream target of TonEBP, PELI1.

Pellino-1 (PELI1) is a E3 ubiquitin ligase, which is part of the Pellino family [18]. According to recent studies, PELI1 mediates NF-κB and MAPK signaling and subsequently modulates the M1-type activation of macrophages [19,20]. In the central nervous system, PELI1 exhibits notable prominence because of its enhanced expression in microglial cells post LPS treatment [21,22]. Additionally, PELI1 is progressively becoming acknowledged as a regulator specific to microglia, contributing to the pathological progression of experimental autoimmune encephalomyelitis (EAE), viral encephalitis, West Nile virus (WNV) encephalitis, and subarachnoid hemorrhage (SHA) [22,23]. Nevertheless, PELI1’s function in ICH is still unresearched, as is its potential association with TonEBP.

Here, we discovered a key transcription factor in microglia and dissected the molecular mechanisms involved in the inflammatory response after ICH. In our study, we carried out a detailed analysis of TonEBP expression in human and mouse brain tissue post-ICH. We further explored the role and mechanism of TonEBP in neuroinflammation. Based on our study, TonEBP enhances LPS-evoked inflammation and NF-κB pathway activation in microglia. Moreover, our findings suggest that TonEBP potentially operates as the transcription factor of PELI1, thereby enhancing the transcriptional activity of the *Peli1* promoter. In conclusion, our study uncovers a previously unidentified downstream target gene of TonEBP, which also represents a promising therapeutic candidate for the improvement of ICH outcomes.

## 2. Results

### 2.1. LPS Evokes Upregulated Transcription Factors in Microglia

**Figure 1:**
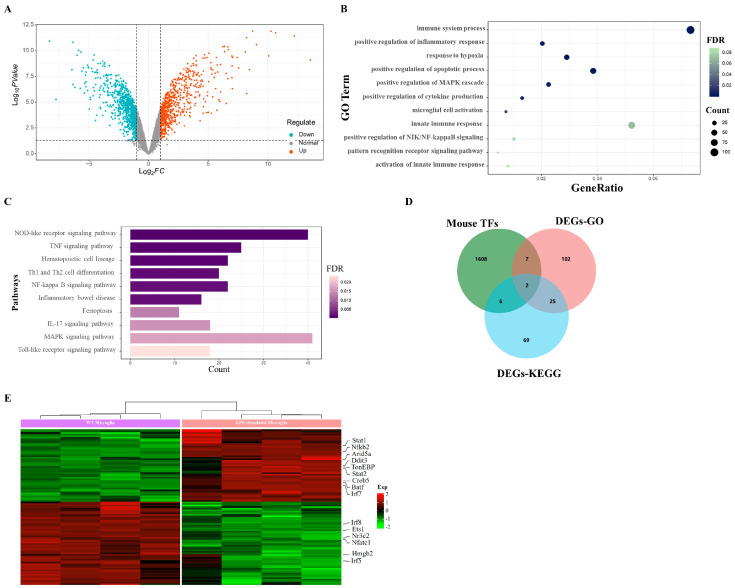
Upregulated transcription factors in the lipopolysaccharide (LPS)-induced inflammatory response in microglia. (**A**) Volcano plot of DEGs at 12 h after lipopolysaccharide (LPS). *P*-adj < 0.05 and |(log2FoldChange)| > 1. (**B**) GO enrichment scatter plot. (**C**) KEGG pathway enrichment analysis plot. (**D**) Wayne diagram showing DEGs-GO and DEGs-KEGG of activated microglia and mouse transcription factors. (**E**) Heat map of different transcription factors in WT microglia and LPS-stimulated microglia.

To elucidate the key genes implicated in neuroinflammation, we analyzed the RNA-seq data derived from GEO (GSE102482). The bioinformatic analysis led to the identification of 683 upregulated and 816 downregulated differentially expressed genes (DEGs) (Figure 1A). For the purpose of understanding the functional pathways linked to these DEGs, we executed a gene ontology (GO) functional analysis and Kyoto Encyclopedia of Genes and Genomes (KEGG) pathway enrichment analysis. The GO functional analysis unveiled significantly enriched biological processes, including the immune system response, positive regulation of inflammation response, cytokine production augmentation, microglial cell activation, positive regulation of NIK/NF-κB signaling, and the pattern recognition receptor signaling pathway (Figure 1B). Furthermore, the KEGG pathway enrichment analysis revealed a robust correlation between these DEGs and inflammation-associated pathways, including the NOD-like receptor signaling pathway, TNF signaling pathway, NF-kappa B signaling pathway, IL-17 signaling pathway, MAPK signaling pathway, and Toll-like receptor signaling pathway (Figure 1C).

In an effort to pinpoint the crucial transcription factors governing the inflammatory response in activated microglia, we intersected these differentially expressed genes (DEGs) with a dataset comprising mouse transcription factors from Animal TFDB4 (hust.edu.cn). This intersection led to the identification of 101 differentially expressed transcription factors (Figure 1D). Out of these, 15 were implicated in inflammation-related biological functions and pathways (Figure 1E). Among the 15 transcription factors, the upregulated transcription factors included signal transducer and activator of transcription 1 (STAT1), nuclear factor kappa B subunit 2 (NFKB2), DNA damage-inducible transcript 3 (DDIT3), tonicity-responsive enhancer-binding protein (TonEBP), signal transducer and activator of transcription 2 (STAT2), CAMP-responsive element-binding protein 5 (CREB5), basic leucine zipper ATF-like transcription factor (BATF), and interferon regulatory factor 7 (IRF7). Conversely, the downregulated transcription factors comprised interferon regulatory factor 8 (IRF8), ETS proto-oncogene 1, ETS proto-oncogene 1, transcription factor (ETS1), nuclear receptor subfamily 3 group C member 2 (NR3C2), nuclear factor of activated T cells 1 (NFATC1), high-mobility group box 2 (HMGB2), and interferon regulatory factor 5 (IRF5) (Figure 1E).

### 2.2. ICH and LPS Cause the Upregulation of Microglial TonEBP

**Figure 2:**
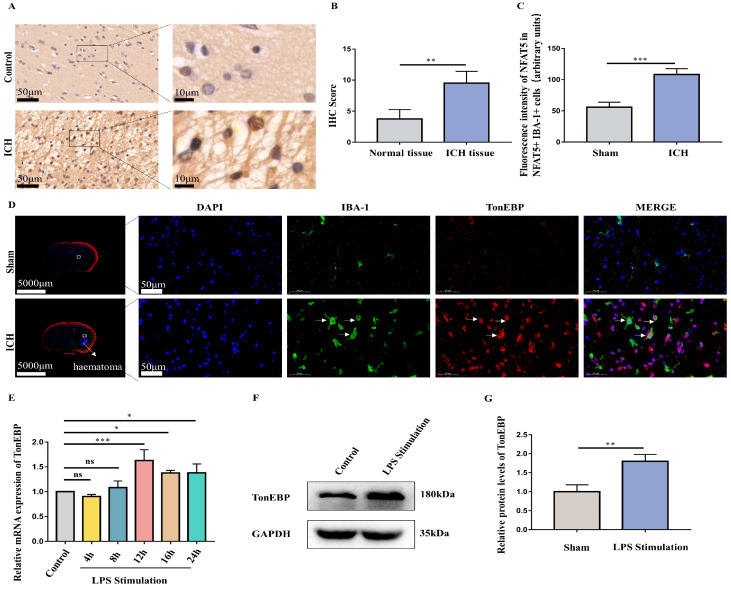
The expression of TonEBP in brain tissues of humans and mice after ICH and in microglia stimulated by LPS. (**A**,**B**) Immunohistochemistry (scale bar = 50 μm or 10 μm) (**A**) and IHC score (**B**) for TonEBP in brain tissues of humans in control group and ICH group. N = 3. (**C**,**D**) Immunofluorescence staining (scale bar = 5000 μm or 50 μm) (**D**) and fluorescence intensity (**C**) of TonEBP and Iba-1in in brain tissues of mice in sham group and ICH group. N = 3. The Iba-1 and TonEBP positive cells were indicated by white arrows. (**E**) RT-PCR of TonEBP in control and 1 μg/mL LPS-treated BV2 cells for different durations (4, 8, 12, 16, and 24 h). N = 3. (**F**,**G**) Western blot of TonEBP in control group and LPS stimulation group. N = 3. The data are presented as mean ± SD. * *p* < 0.05, ** *p* < 0.01, *** *p* < 0.001, ns: non significant.

Considering that the ICH triggered neuroinflammation, particularly in near-hematoma regions, we conducted immunohistochemistry on control tissue and post-ICH tissue in the human brain. Among the various transcription factors examined, our findings revealed an enhanced TonEBP in the peri-hematoma region of the human brain post-ICH (Figure 2A,B). Furthermore, we established ICH models in mice and collected their brain tissue 3 days after the establishment of the models. Microglia, labeled in green with Iba-1, and TonEBP, labeled in red, were examined using immunofluorescence. It was found that TonEBP was co-localized with Iba-1, and that the TonEBP fluorescence intensity in Iba-1-positive cells was increased after ICH (Figure 2C,D). In order to simulate the inflammation in microglia, we stimulated the BV2 with 1 μg/mL concentrations of LPS. Furthermore, BV2 were treated with LPS for different durations (4, 8, 12, 16, and 24 h). It was found that LPS needed to be sustained for at least 12 h to cause a sustained and stable elevation in TonEBP (Figure 2E). Additionally, our Western blot results demonstrated that the protein levels of TonEBP in BV2 were elevated under the 1 μg/mL LPS treatment for 12 h (Figure 2F,G).

### 2.3. TonEBP Exacerbates Neuroinflammation and Microglial Activation

**Figure 3:**
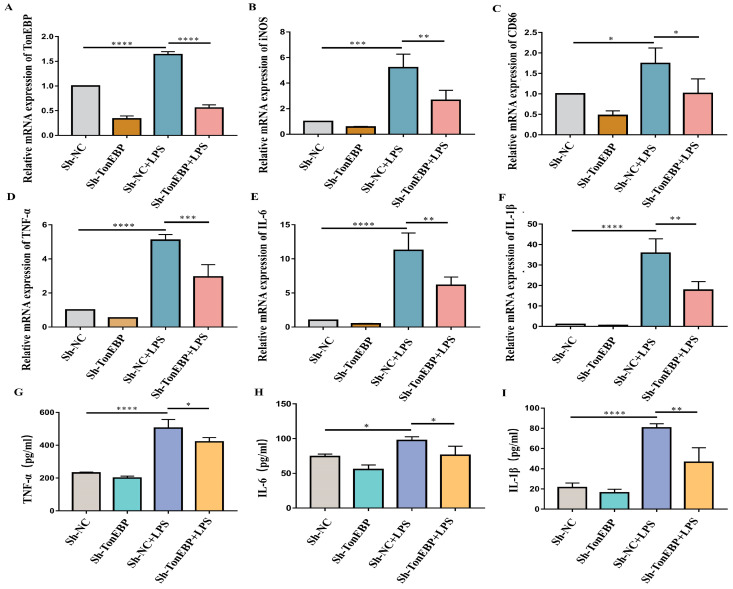
Inflammation and microglial activation were ameliorated by TonEBP deficiency. (**A**–**C**) RT-PCR of TonEBP (**A**), iNOS (**B**), and CD86 (**C**) in sh-NC, sh-TonEBP, sh-NC+LPS, and sh-TonEBP+LPS groups. N = 3. (**D**–**F**) RT-PCR of TNF-α (**D**), IL-6 (**E**), and IL-1β (**F**) in sh-NC, sh-TonEBP, sh-NC+LPS, and sh-TonEBP+LPS groups. N = 3. (**G**–**I**) ELISA of TNF-α (**D**), IL-6 (**E**), and IL-1β (**F**) in sh-NC, sh-TonEBP, sh-NC+LPS, and sh-TonEBP+LPS groups. N = 3. The data are presented as mean ± SD. * *p* < 0.05, ** *p* < 0.01, *** *p* < 0.001, **** *p* < 0.0001.

To demonstrate the influence of TonEBP on neuroinflammation and microglia activation upon LPS stimulation, we generated TonEBP-knockdown cells using sh-RNA and evaluated the efficiency of TonEBP knockdown with RT-PCR assays (Figure 3A). Our RT-PCR results demonstrated a successful knockdown of TonEBP expression in BV2 cells. Additionally, RT-qPCR assays were conducted to measure the mRNA levels of the pro-inflammatory cytokines TNF-α, IL-6, and IL-1β. The findings indicated that TonEBP deficiency in BV2 cells remarkably attenuated the mRNA of TNF-α, IL-6, and IL-1β after LPS treatment (Figure 3D–F). Moreover, a medium from BV2 was also obtained to evaluate the protein levels of TNF-α, IL-6, and IL-1β using ELISA assays. LPS treatment increased the protein levels of TNF-α, IL-6, and IL-1β in the medium from BV2, while TonEBP deficiency reduced the protein levels of these cytokines elicited by LPS stimulation (Figure 3G–I). Furthermore, the pro-inflammatory microglia markers iNOS (nitric oxide synthase 2) and CD86 (CD86 molecule) were elevated upon LPS stimulation, while TonEBP deficiency ameliorated the elevation of iNOS and CD86 caused by LPS stimulation (Figure 3B,C). These results indicate that TonEBP deficiency reduces the mRNA and protein levels of pro-inflammatory cytokines and ameliorates microglia activation in response to LPS stimulation.

### 2.4. TonEBP Contributes to the Activation of the NF-κB Signaling Pathway

**Figure 4:**
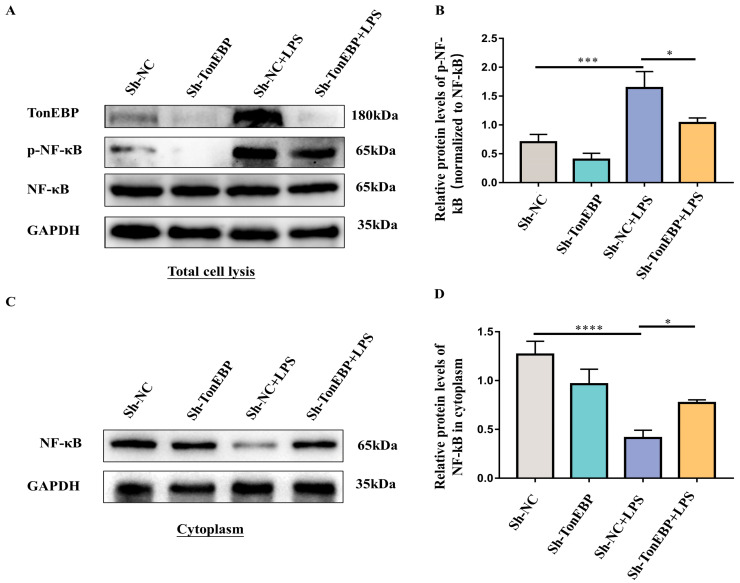
TonEBP deficiency reduced the activation of the NF-κB signaling pathway. (**A**,**B**) Western blot of TonEBP, p-NF-κB, and NF-κB of total cell lysis in sh-NC, sh-TonEBP, sh-NC+LPS, and sh-TonEBP+LPS groups. N = 3. (**C**,**D**) Western blot of NF-κB of cytoplasm in sh-NC, sh-TonEBP, sh-NC+LPS, and sh-TonEBP+LPS groups. N = 3. The data are presented as mean ± SD. * *p* < 0.05, *** *p* < 0.001, **** *p* < 0.0001.

Researchers have reported that the NF-κB pathway is essential for pro-inflammatory cytokine secretion. NF-κB signaling is triggered by DAMPs (damage-associated molecular patterns), promoting the shifting of the RELA/p65-NFKB1/p50 heterodimeric complex from the cytoplasm to the nucleus. After translocation to the nucleus, this complex binds to chromatin and subsequently activates the production of various pro-inflammatory cytokines, including TNF-α, IL-6, and IL-1β. To more deeply investigate the function of TonEBP in the modulation of NF-κB signaling, we assessed the phosphorylation of the RELA/p65 subunits of NF-κB. Western blot results demonstrated upregulation in the phosphorylation of RELA/p65, whereas the knockdown of TonEBP by sh-RNA decreased the phosphorylation of RELA/p65 under LPS stimulation in BV2 (Figure 4A,B). Additionally, LPS treatment decreased cytoplasmic NF-κB, while TonEBP deficiency mitigated this decrease (Figure 4C,D). These results demonstrate that TonEBP facilitates the activation of NF-κB signaling.

### 2.5. RNA-seq for Potential Targets of TonEBP 

**Figure 5:**
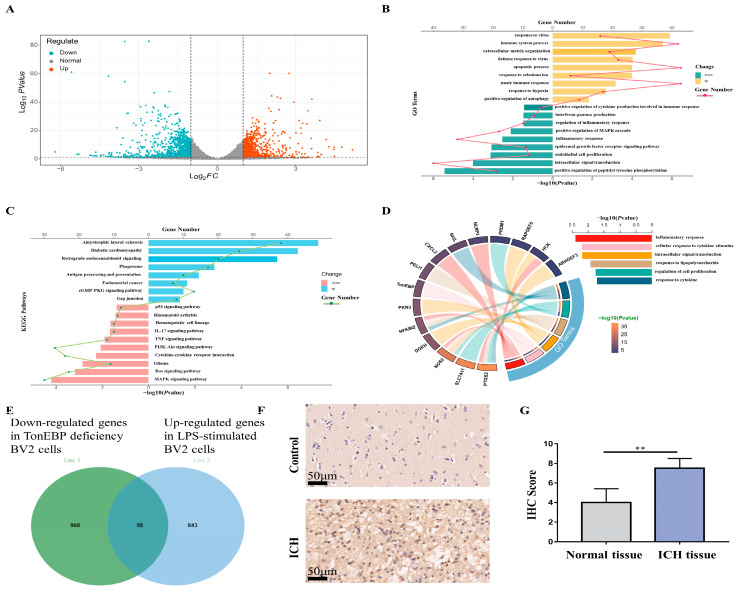
RNA-seq for potential target of TonEBP. (**A**) Volcano plot of DEGs between the sh-NC group and sh-TonEBP group. *p*-value < 0.05 and |(log2FoldChange)| > 1. (**B**) GO enrichment scatter plot. (**C**) KEGG pathway enrichment analysis plot. (**D**) GO enrichment chord diagram and bar plot legend for GO terms. (**E**) Wayne diagram showing upregulated DEGs in LPS-stimulated BV2 cells and downregulated DEGs in TonEBP-deficient BV2 cells. (**F**,**G**) Immunohistochemistry (scale bar = 50 μm) (**F**) and IHC score (**G**) for PELI1 in brain tissues of humans in control group and ICH group. N = 3. The data are presented as mean ± SD. ** *p* < 0.01.

Next, for the purpose of exploring downstream of TonEBP, we conducted RNA-seq analysis on control and TonEBP-deficient BV2. The volcano plot of the RNA-seq analysis revealed 779 upregulated genes and 1026 downregulated genes in the sh-TonEBP BV2 cells compared to the sh-NC cells (Figure 5A). Subsequently, we performed GO function analysis and KEGG pathway enrichment analysis on the differentially expressed genes (DEGs). The bar plot displayed the significantly enriched GO terms in the upregulated and downregulated DEGs. The increased genes were notably clustered in biological processes such as the response to virus, immune system processes, extracellular matrix organization, the defense response to virus, and apoptotic processes. Nevertheless, the downregulated genes were prominently enriched in processes such as the positive regulation of peptidyl-tyrosine phosphorylation, intracellular signal transduction, the inflammatory response, positive regulation of MAPK cascade, and the positive regulation of cytokine production involved in the immune response (Figure 5B). The subsequent bar plot illustrated the prominently enriched KEGG pathways. The upregulated DEGs were involved in pathways such as amyotrophic lateral sclerosis, diabetic cardiomyopathy, retrograde endocannabinoid signaling, phagosome, antigen processing and presentation, and the cGMP-PKG signaling pathway. Conversely, the downregulated DEGs were mainly associated with pathways such as the MAPK signaling pathway, Ras signaling pathway, cytokine–cytokine receptor interaction, TNF signaling pathway, and IL-17 signaling pathway (Figure 5C). These results indicate significant changes in the inflammation-related biological functions and pathways in the downregulated DEGs.

To further investigate the potential target of TonEBP, we conducted an analysis by comparing the downregulated genes in TonEBP-deficient BV2 cells with the upregulated genes in LPS-stimulated BV2 cells. This analysis revealed 58 genes that were found to be common to both conditions (Figure 5E). Subsequently, we performed a functional analysis of these 58 genes using GO analysis to identify the genes associated with inflammatory immunity. As a result, we identified 15 genes that are involved in various inflammatory processes, including the inflammatory response (PTGS, NOS2, NFKBIZ, CXCL2, NLRP3, and HCK), the cellular response to cytokine stimulus (TonEBP), intracellular signal transduction (DGKH, PKN3, RAPGEF5, and ARHGEF3), the response to lipopolysaccharide (PELI1), the regulation of cell proliferation (SLC7A11 and PRDM1), and the response to cytokine (SKIL) (Figure 5D). Notably, our immunohistochemical staining results demonstrated an enhanced expression of PELI1 in human brain tissue with ICH (Figure 5F,G).

### 2.6. TonEBP Regulates PELI1 Expression at the Transcriptional Level

**Figure 6:**
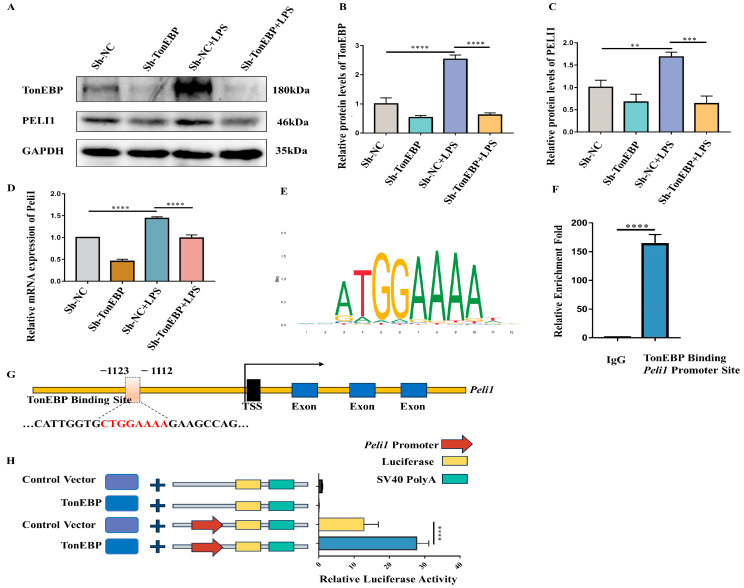
TonEBP acted as a transcription factor of PELI1. (**A**–**C**) Western blot of TonEBP (**A**,**B**) and PELI1 (**A**,**C**) in sh-NC, sh-TonEBP, sh-NC+LPS, and sh-TonEBP+LPS groups. N = 3. (**D**) RT-PCR of PELI1 (**D**) in sh-NC, sh-TonEBP, sh-NC+LPS, and sh-TonEBP+LPS groups. N = 3. (**E**) JASPAR-derived TonEBP chromatin binding motif. (**F**) CUT & RUN experiment for TonEBP and *Peli1* promoter. N = 3. (**G**) Scheme of TonEBP binding sequence in *Peli1* promoter. (**H**) The dual-luciferase reporter assay of TonEBP and *Peli1* promoter. N = 7. ** *p* < 0.01, *** *p* < 0.001, **** *p* < 0.0001.

Next, we began to explore the possibility that PELI1 is downstream of TonEBP. Firstly, we performed a Western blot analysis to examine PELI1 in TonEBP-deficient BV2 under LPS stimulation. The results revealed that TonEBP and PELI1 were enhanced after LPS stimulation in sh-NC BV2 cells, while TonEBP and PELI1 expression decreased in TonEBP-deficient cells under LPS stimulation. (Figure 6A–C). To assess whether TonEBP regulates the mRNA level of PELI1 under LPS stimulation, we conducted RT-PCR to measure the mRNA levels of PELI1. The RT-PCR showed that TonEBP deficiency reduced the mRNA of PELI under LPS stimulation (Figure 6D). Next, we asked whether TonEBP regulates the transcription of *Peli1*. To address this question, we predicted the potential binding site of TonEBP to the *Peli1* promoter region using the JASPAR website. The analysis showed the presence of a potential binding site upstream of the *Peli1* transcription start site (Figure 6E,G). Subsequently, we performed a CUT & RUN experiment to verify the interaction between TonEBP and the *Peli1* promoter region. The results of the CUT & RUN experiment indicated a strong interaction between TonEBP and the *Peli1* promoter region 1123 to 1112, upstream of the transcription start site of the *Peli1* gene. (Figure 6F). Furthermore, to substantiate whether NAFT5 would promote the transcription activity of *Peli1*, we conducted a dual-luciferase reporter experiment. The dual-luciferase reporter assay results indicated that TonEBP strongly promoted the transcription activity of the *Peli1* gene. (Figure 6H). These findings suggest that TonEBP interacts with the *Peli1* promoter region and promotes *Peli1* transcriptional activity, leading to elevated PELI1 expression, which ultimately regulates inflammation in BV2 cells.

## 3. Discussion

Numerous studies have provided compelling evidence that transcription factors (TFs) are instrumental in modulating microglial functions across a variety of biological processes, including microglial activation [8,24], such as the NFE2-like BZIP transcription factor 2 (Nrf2), activating transcription factor 4 (ATF4), and signal transducer and activator of transcription family (STATs) [25,26,27]. Here, we report a potential transcription factor, TonEBP, which may regulate microglial activation. Some studies have reported abnormal TonEBP expression in the lesion regions of various diseases. Laban et al. published a study stating that hypoxia increased TonEBP levels in the lung and cultured smooth muscle cells within 24 h [28]. In addition, Shin et al. reported enhanced TonEBP expression in the brains of a kainic-acid-induced mouse seizure model [29]. Our current work suggests increased TonEBP expression within the peri-hematoma region of the human brain following ICH. Furthermore, our immunofluorescence experiments also demonstrated the specific co-localization of TonEBP with the microglial marker Iba-1 and elevated TonEBP fluorescence intensity in Iba-1-positive microglial cells in post-ICH mice compared to the sham group. These findings indicated elevated TonEBP in the human hemorrhagic brain and in the microglia of post-ICH mouse brains. 

In further in vitro experiments, we observed that microglial TonEBP is triggered by LPS cellular stimulation. Moreover, a deficiency in microglial TonEBP mitigated the induction of pro-inflammatory cytokines like TNF-α, IL-6, and IL-1β. This deficiency also inhibited the expression of the activated microglial markers iNOS and CD86. Our findings align with previous research on macrophages, in that elevated TonEBP propels the differentiation of macrophages into the M1 phenotype when responding to inflammatory signals, including LPS or hyperglycemia [30,31]. Taken together, our results suggest that TonEBP may function as a key transcription factor in microglia activation and the microglia-mediated inflammation response. Nevertheless, due to the limitations of in vivo experiments, it is difficult to evaluate whether TonEBP participates in neurological deficits in an in vivo ICH mouse model. Therefore, additional research and experimental studies are imperative to clarify the function of TonEBP in ICH.

The substantial reduction in pro-inflammatory cytokines and the microglia activation in LPS-stimulated TonEBP-deficient BV2 led us to hypothesize that the inflammation regulatory role of TonEBP might be linked to NF-κB signaling pathways. It is widely recognized that nuclear factor kappa B (NF-κB) controls the majority of activated microglia cell signature genes in central nervous system diseases [32]. However, the correlation between TonEBP and NF-κB has been a subject of debate. Some studies have reported that TonEBP regulates the phosphorylation of NF-κB and subsequent NF-κB signaling pathway activation [33,34,35]. However, other findings have revealed a connection between TonEBP and the NF-κB complex via a different mechanism, suggesting that TonEBP enhances the transcriptional activity of NF-κB in response to LPS by acting as a cofactor for NF-κB [11,30,36]. Our results align with former studies stating that TonEBP deficiency significantly diminishes the phosphorylation of p65, a crucial NF-κB complex, in LPS-stimulated BV2 cells. Moreover, our results also indicate that TonEBP deficiency in LPS-stimulated BV2 cells increases the levels of the cytoplasmic p65 protein, suggesting that TonEBP regulates NF-κB phosphorylation and its subsequent translocation.

The RNA-seq analysis we conducted provided us with a more profound understanding through which to explore the regulation of NF-κB activation in microglia cells by TonEBP. The gene ontology (GO) biological enrichment process identified 15 potential inflammation-related genes targeted by TonEBP. Among these genes, NLRP3 (NLR family pyrin domain-containing 3), CXCL2 (C-X-C motif chemokine ligand 2), and NOS2 (nitric oxide synthase, inducible) have been reported to recruit TonEBP to their promoter regions, thereby enhancing transcriptional activity [37,38,39]. These findings led us to hypothesize that the new downstream gene of TonEBP could be among these 15 potential target genes. Intriguingly, the detection of upregulated PELI1 expression in the peri-hematoma region of ICH human brain provided us with a direction for further investigation into the relationship between TonEBP and PELI1. Emerging evidence suggests that PELI1 plays a crucial role in the inflammation regulation in immune cells, including T cells, macrophages, and microglia, across multiple diseases [40,41]. In the central nervous system, due to the cell specificity of PLEI1 to microglia, the critical inflammation regulatory role of PELI1 is primarily focused on microglia [21,22,42]. Mechanistically, PELI1 regulates the K63-ubiquitination of IRAK1 (interleukin-1 receptor-associated kinase 1) and Traf3/Traf6 (TNF receptor-associated factor 3,6), facilitating subsequent NF-κB activation [21,22,43,44]. As anticipated, our research revealed that PELI1 was upregulated in LPS-stimulated BV2 cells. Moreover, TonEBP knockdown significantly reduced PELI1 expression at both the protein and mRNA levels. Furthermore, the interaction between TonEBP and the *Peli1* promoter and the enhanced *Peli1* transcriptional activity by TonEBP suggest that PELI1 is a direct target of TonEBP. In summary, our findings suggest that TonEBP may regulate NF-κB activation by transcriptionally modulating PELI1. 

## 4. Materials and Methods

### 4.1. Human Tissues

This study was approved by the Ethics Committee of the Children’s Hospital of Chongqing Medical University (File No. 2021295) and was conducted in compliance with ethical standards and the Declaration of Helsinki. Brain tissues were obtained from patients with spontaneous intracerebral hemorrhage (n = 3 per group). Control tissues were provided by the Department of Forensic Medicine, Chongqing Medical University (n = 3, per group). The details are shown in Supplementary Table S1. 

### 4.2. Animals

The Institutional Animal Ethics Committee of Chongqing Medical University sanctioned all animal care and procedures. Male C57BL/6 mice, aged between 8 and 10 weeks (n = 3 per group), were procured from the Laboratory Animal Center of the same university. The mice were housed under a standard light/dark cycle and were also provided with unrestricted access to nourishment and hydration.

### 4.3. ICH Model

The induction of the murine intracerebral hemorrhage (ICH) model was achieved through the stereotactic-guided delivery of bacterial collagenase VII (C0773, Sigma-Aldrich, St. Louis, MO, USA) into the right basal ganglia [45]. Initially, mice were anesthetized with an intraperitoneal administration of pentobarbital (40 mg/kg) and subsequently positioned within a stereotaxic frame (RWD Life Science, Shenzhen, China). Following this, the bacterial collagenase VII (0.1 U in 1 μL) was administered at 0.2 mm posterior and 2.2 mm lateral of the bregma, 3.5 mm in depth, at a speed of 0.1 μL/min. The burr hole was subsequently closed with sterilized medical bone wax after the syringe remained in place for 10 min post administration. For the sham mice, an identical procedure was conducted with the replacement of bacterial collagens with saline. Throughout both the experimental and recovery periods, the animals’ body temperatures were maintained at 37.0 ± 0.5 °C.

### 4.4. Cell Culture and LPS Treatment

The BV-2 cell, a widely utilized murine microglia cell line (National Infrastructure of Cell Line Resource, Beijing, China), was cultivated in Dulbecco’s Modified Eagle Medium (Gibco, Thermo Fisher Scientific, New York, NY, USA) supplimented with 10% FBS (VivaCell, Shanghai, China), 100 U/mL penicillin, and 100 g/mL streptomycin (15140122, Gibco, Thermo Fisher Scientific, New York, USA). The cells were maintained in a humidified atmosphere with 5% CO_2_ at a temperature of 37 °C. For LPS stimulation, BV2 cells were treated with 1 μg/mL LPS [46] for the indicated time.

### 4.5. RNA Extraction and Real-Time Quantitative PCR

RNA was isolated utilizing the TRIZOL reagent, adhering to the protocol provided by the manufacturer (Invitrogen, San Diego, CA, USA). Subsequently, a volume of 800 nanograms of RNA was reversed into cDNA via the ABScript III RT Master Mix (RK20429, ABclonal, Wuhan, China). The quantitative PCR process was executed utilizing the SYBR Green Fast qPCR Mix (RK21203, ABclonal, Wuhan, China). The relative expression was quantified via the 2-ΔΔCT method, which underwent normalization to GAPDH. The primers used in the article are shown in Supplementary Table S2. 

### 4.6. ELISA Assay for Cytokines

Enzyme-linked immunosorbent assay (ELISA) kits (Jiubang, Quanzhou, China) were used to determine the levels of TNF-α (QZ-10225), IL-6 (QZ-10260), and IL-1β (QZ-10247) in the treated BV2 cell culture medium as per the manufacturer’s instructions.

### 4.7. Immunofluorescence

Mice were euthanized and sacrificed 72 h post-ICH model induction. The mouse brains were fixed with a 4% paraformaldehyde solution prior to being sectioned into 8 μm slices. These sections were permeabilized and blocked with a 5% goat serum solution containing 0.5% Triton X-100. Next, the section was incubated with the indicated primary antibody at 4 °C overnight, followed by treatment with the appropriate secondary antibody for 1 h at 37 °C. Finally, the sections were subjected to incubation with DAPI and observation with a fluorescence microscope. The antibodies used for immunofluorescence were rabbit anti-TonEBP (ab3446, Abcam, Waltham, MA, USA) and anti-Iba-1 (GB15105-100, Servicebio, Wuhan, China).

### 4.8. Immunohistochemical Staining

The paraffin-embedded human brain tissue sections underwent deparaffinization and rehydration prior to antigen retrieval. Subsequently, the brain sections were incubated with the indicated primary and secondary antibodies after the inhibition of internal peroxidase activity. A DAB substrate kit (ZLI-9018; ZSGB, Beijing, China) was employed for visualization. Lastly, the brain tissue was counterstained with hematoxylin and sealed. The immunostaining intensity was scored as follows: 0 (no immunostaining), 1 (weak immunostaining), 2 (moderate immunostaining), or 3 (strong immunostaining). The percentage of positive cells was scored as 0 (<5%), 1 (5–25%), 2 (26–50%), 3 (51–75%), or 4 (76–100%). Finally, the immunostaining intensity score was multiplied by the percentage-of-positive-cells score to calculate each sample’s score. The antibodies used for immunohistochemical staining were rabbit anti-TonEBP (ab3446, Abcam, Waltham, USA) and rabbit anti-PELI1 (ab199336, Abcam, Waltham, USA).

### 4.9. Construction and Transfection of Plasmids

Full-length sequences of TonEBP were procured from the cDNA of the BV2 cell and subsequently cloned into the Plenti6/TR vector. The *Peli1* promoter region was synthesized by BGI (Beijing Genomics Institute, Beijing, China). Plasmids were transiently transfected into HEK293T cells with Linea Polyethylenimine (PEI) in accordance with the manufacturer’s protocol (polyscience, Niles, IL, USA). For BV2 cells, the scrambled sh-RNA and TonEBP lentivirus vector were constructed by OBiO (Shanghai, China) and transfected to the BV2 cells. The primers used for the plasmid construction and sh-RNA sequence are shown in Supplementary Table S3.

### 4.10. Promoter Luciferase Reporter Assay

The promoter region of the *Peli1* gene, extending from the −2000 base site to the transcription start site, was integrated into the modified pGL4.10 vector (Promega, Madison, WI, USA). Full-length sequences of TonEBP were extracted from the cDNA of the BV2 cell and cloned into the Plenti6/TR vector. For plasmid transfection, HEK293T cells were cultured on 24-well plates and transfected with indicated plasmids 48 h before harvesting. The activity of the Firefly luciferase and the signal of the Renilla luciferase were quantified using the dual-luciferase reporter assay kit (Promega, Madison, USA) and detected using a GloMax 20/20 Luminometer (Promega).

### 4.11. Cleavage under Targets and Release Using Nuclease (CUT&RUN) Assay

The interaction between protein and DNA was ascertained using the hyperactive pG-MNase CUT & RUN assay kit for PCR/qPCR (Vazyme, HD101, Nanjing, China). Initially, the potential binding site of TonEBP on the *Peli1* promoter was predicted using the JASPAR website [47]; the primers were designed based on this predicted binding site. For the CUT & RUN assay, 3 × 10^5^ BV2 cells were harvested and incubated with pre-treated ConA Beads Pro for 10 min at room temperature. Following this, 6 ug of antibodies were added to the cell–bead crosslink and incubated overnight at 4 °C. Subsequently, CaCl2 was added to initiate cleavage after the pG-MNase enzyme was linked to the antibody–cell–bead crosslink. Finally, the released DNA fragments were collected and quantified using RT-qRCR with ChamQ Universal SYBR qPCR Master Mix. The primers were forward primer TGGGCATCCAGGGTTTTTGA and reverse primer GGTAATTTGCCTCAGTTTCCCT.

### 4.12. RNA Sequencing and Analysis

For RNA-seq analysis, total RNA was extracted from BV2 cells with a TRIzolKit (Invitrogen, California, USA). RNA-seq library preparation and subsequent RNA-seq sequencing were conducted using SHANGHAI BIOPROFILE (Shanghai, China), in which the prepared library was sequenced on the Illumina (San Diego, CA, USA) Novaseq6000 platform. The RNA-seq data were uploaded to the GEO database under accession number GSE249968. The RNA-seq data on LPS-stimulated primary microglia and the microarray data on LPS-stimulated BV2 cells were obtained from the GEO database under accession numbers GSE102482 and GSE103156, respectively.

For data processing and analysis, raw counts were used to analyze differentially expressed genes (DEGs) using the DESeq2 R package, with cutoffs at log2 fold change > 1 or < −1 and *p*-value < 0.05. GO (gene ontology) and KEGG (Kyoto Encyclopedia of Genes and Genomes) analyses were performed on the DAVID website [48,49], and the results were visualized in R (4.2.2). The DEGs were compared using the website tool Jvenn [50].

### 4.13. Lentivirus Transfection

OBiO Technology (Shanghai, China) engineered the lentivirus. The pSLenti-puro vector was utilized to clone both the silencing and non-silencing shRNA sequences of murine TonEBP, which were subsequently packaged into the lentivirus. Following this, BV2 cells underwent a 14 h transfection process with the lentivirus. A selection process was implemented 72 h post-infection, utilizing 2 μg/mL of puromycin (T19978, TargetMol, Boston, MA, USA). 

### 4.14. Western Blot

Protein extraction was performed on BV2 and mouse brain samples using RIPA lysis buffer (P0013B, Beyotime, Shanghai, China) supplemented with a 1 mM protease inhibitor cocktail (B14001, Selleck, Houston, TX, USA). The lysates were then centrifuged to gather supernatants. Nuclear and cytoplasmic proteins were extracted using a cytoplasmic and nuclear protein extraction kit (AR0106, Boster, Wuhan, China). The proteins were transferred onto a PVDF membrane (Millipore, Shanghai, China) after being resolved using suitable SDS-PAGE. The membrane was blocked in 5% non-fat milk solution for one hour at room temperature, then incubated overnight at 4 °C with primary antibodies. Immunoblotting was identified using horseradish peroxidase-linked anti-rabbit or anti-mouse secondary antibodies, in conjunction with the BeyoECL Moon super sensitivity detection kit (Beyotime, Shanghai, China). The antibodies and concentrations used for Western blotting were as follows: rabbit anti-TonEBP (ab3446, Abcam, Waltham, USA) 1:1000, rabbit anti-NF-κB p65 (8242, Cell Signaling Technology, Danvers, MA, USA) 1:2000, rabbit anti-phospho-NF-κB p65 (Ser536) (3033, Cell Signaling Technology, Danvers, USA) 1:500, mouse anti-PELI1 (TA807629S, ORIGENE, Rockville, MD, USA) 1:2000, and mouse anti-GAPDH (60004-1-Ig, Proteintech, Wuhan, China) 1:50,000.

### 4.15. Statistical Analysis

Data are presented as mean ± SD. GraphPad Prism software (version 8.0) was used for statistical analyses. Two groups were analyzed using Student’s *t*-test. One-way ANOVA and Tukey’s multiple comparison test were used to analyze data between more than two groups. Differences between means were considered statistically significant when *p* < 0.05.

## 5. Conclusions

In summary, our current research investigates an increased transcription factor, TonEBP, after hemorrhagic stroke in the peri-hematoma region of the brain in humans and mice. Furthermore, our in vitro experiment shows that TonEBP may act as a transcription factor of PELI1, which has been reported to be a regulator of the NF-κB pathway. Moreover, we demonstrate that the knockdown of microglial TonEBP could regulate the NF-κB pathway and attenuate the inflammation response. Taken together, TonEBP may mediate the regulation of the NF-κB pathway and the inflammatory response via promoting the expression of PELI1 transcriptionally. Our research may provide a potential therapeutic target for intracerebral hemorrhage and elucidate part of the underlying mechanism of TonEBP in the inflammatory response. However, the role of microglial TonEBP in neuroinflammation after a hemorrhagic stroke in mice deserves our further attention.

## Data Availability

Data are contained within the article and Supplementary Materials.

## Supplementary Materials

The following supporting information can be downloaded at: https://www.mdpi.com/article/10.3390/ijms25031438/s1. Supplementary Table S1: Human tissue information. Supplementary Table S2: Primer sequences for RT-qPCR, Supplementary Table S3: Sh-RNA sequences and primer sequences for plasmids construction.
