# Supplementary material for: TonEBP: A Key Transcription Factor in Microglia Following Intracerebral Hemorrhage Induced-Neuroinflammation
Source: Int J Mol Sci. 2024 Jan 24;25(3):1438. doi: 10.3390/ijms25031438 (PMC10855931; doi:10.3390/ijms25031438)
Supplement: Supplementary file 1 [file ijms-25-01438-s001.zip › ijms-2784103-supplementary.pdf]

Additional file

Supplementary Table S1. Human tissue information

Sample	sex	age	Possible bleeding causes	ICH location	Estimated ICH volume	Performed experiment
ICH 1	M	11	Arteriovenous malformation	Left parietal-occipital lobe	40mL	IHC
ICH 2	F	11	Cerebrovascular malformation	Left frontotemporal lobe	30mL	IHC
ICH 3	F	10	Arteriovenous malformation	Midline area, left parietal lobe	30mL	IHC
CTR1	M	New-born	-	-	-	IHC
CTR2	M	59	-	-	-	IHC
CTR3	F	60	-	-	-	IHC

Supplementary Table S2. Primer sequences for RT-qPCR

Genes	Forward Primer（5′—3′）	Reverse Primer（5′—3′）
TonEBP	AGCAGCCTCCAATCTCACAC	GGGCGCTGTGTTTGGAAAAT
TNF-α	CTCCAGGCGGTGCCTATGTC	CCATTTGGGAACTTCTCATCCCTTT
IL-1β	AGCTTCCTTGTGCAAGTGTC	TTGGGGTCCGTCAACTTCAA
IL-6	GTTCCTCTCTGCAAGAGACTTC	AGTCTCCTCTCCGGACTTGT
iNOS	AATCTTGGAGCGAGTTGTGG	CAGGAAGTAGGTGAGGGCTTG
CD86	CTCCCACCACAAATGGCACT	CTTGGACCTTGGACTAGGCG
PELI1	GCAACAAGGACCAGCATAGC	TACTGACTGCGTGTCGGAAT
GAPDH	GATGCAGGGATGATGTTCTG	GTGAAGGTCGGTAACGG

Supplementary Table S3. Sh-RNA sequences and primer sequences for plasmids construction

Oligonucleotides	Forward primer（5′—3′）	Feverse primer（5′—3′）
TonEBP	GCTCTAGAATGCCCTCGGACTTCATCTCA	CCGCTCGAGAAAGGAGCCGGTTAAATTGTTCC
Scrambled sh-RNA	UUC UCC GAA CGU GUC ACG UTT	ACG UGA CAC GUU CGG AGA ATT
TonEBP sh-RNA	CCAGUUCCUACAAUGAUAACACU	AGUGUUAUCAUUGUAGGAACUGG
